# FROM FEAR TO FORTITUDE AND EMPOWERMENT: A PATIENT’S JOURNEY

**DOI:** 10.1093/geroni/igae098.1704

**Published:** 2024-12-31

**Authors:** Patricia Nece

**Affiliations:** Obesity Action Coalition, Alexandria, Virginia, United States

## Abstract

Many older adults aged 65 and over with overweight and obesity struggle with navigating a health care system that is not inherently equipped to deliver quality obesity care. In the face of ageism and weight stigma, initiating conversations with providers who lack time, education, training, or even empathy can test the “fortitude factor” of even the most resilient of patients. Patricia Nece, J.D. will share her own personal experiences of such challenges and how the Obesity Bill of Rights can empower other patients facing similar situations to advocate for themselves—especially when it comes to making decisions about their own health care.

